# CircRNA signature predicts immunotherapy response in advanced non-small cell lung cancer

**DOI:** 10.1177/17588359251395920

**Published:** 2025-11-25

**Authors:** Xin Li, Shixiang Wang, Yanru Cui, Su-Han Jin, Junzhu Xu, Chi Zhang, Juanyan Shen, Hu Ma, Jian-Guo Zhou

**Affiliations:** Department of Oncology, The Second Affiliated Hospital of Zunyi Medical University, Zunyi, P.R. China; Key Laboratory for Cancer Prevention and treatment of Guizhou Province, Zunyi, P.R. China; Department of Biomedical Informatics, School of Life Sciences, Central South University, Changsha, P.R. China; Meinig School of Biomedical Engineering, Cornell University, Ithaca, NY, USA; Department of Orthodontics, Affiliated Stomatological Hospital of Zunyi Medical University, Zunyi, P.R. China; Department of Oncology, The Second Affiliated Hospital of Zunyi Medical University, Zunyi, P.R. China; Key Laboratory for Cancer Prevention and treatment of Guizhou Province, Zunyi, P.R. China; Department of Oncology, The Second Affiliated Hospital of Zunyi Medical University, Zunyi, P.R. China; Key Laboratory for Cancer Prevention and treatment of Guizhou Province, Zunyi, P.R. China; Department of Oncology, The Second Affiliated Hospital of Zunyi Medical University, Zunyi, P.R. China; Key Laboratory for Cancer Prevention and treatment of Guizhou Province, Zunyi, P.R. China; Department of Oncology, The Second Affiliated Hospital of Zunyi Medical University, Zunyi, Guizhou 563000, P.R. China; Key Laboratory for Cancer Prevention and Treatment of Guizhou Province, Zunyi, P.R. China; Department of Oncology, The Second Affiliated Hospital of Zunyi Medical University, Zunyi, Guizhou 563000, P.R. China; Key Laboratory for Cancer Prevention and Treatment of Guizhou Province, Zunyi, P.R. China

**Keywords:** atezolizumab, circular RNAs, non-small cell lung cancer, predictive biomarker

## Abstract

**Background:**

Immune checkpoint inhibitors (ICIs) offer significant benefits for advanced non-small cell lung cancer (NSCLC) but yield objective response rates of only 10%–30% in unselected patients. Circular RNAs (circRNAs), implicated in cancer RNA dysregulation, may serve as biomarkers for ICI response.

**Objectives:**

Identify circRNA signature to predict atezolizumab efficacy of NSCLC.

**Design:**

This study analyzed circRNA expression profiles from 891 advanced NSCLC patients in the OAK and POPLAR clinical studies.

**Methods:**

Based on The Cancer CircRNA Immunome Atlas database, we identified circRNAs associated with the efficacy of immunotherapy in NSCLC patients. Then, we establish predictive models for immunotherapy efficacy using multiple methods and conduct performance verification. Finally, we performed Gene Set Enrichment Analysis and Gene Set Variation Analysis to explore potential mechanisms.

**Results:**

We identified an 11-circRNA signature, named circRNA-Sig, which predicted atezolizumab efficacy with an area under the curve of 0.71 in OAK and 0.67 in POPLAR. Survival analysis in OAK showed patients with low circRNA-Sig scores benefited more from ICI than chemotherapy (hazard ratio (HR) = 1.347; 95% confidence interval (CI): 1.049–1.730; *p* = 0.019), whereas those with high scores showed no significant difference (HR = 1.020; 95% CI: 0.796–1.307; *p* = 0.876). Enrichment analysis revealed that low-scoring patients exhibit an activated tumor immune microenvironment, with upregulated pathways in interferon-γ and IL-2/STAT5, which can activate immune cells such as CD8 + T cells and natural killer cells, suggesting mechanistic links to ICI sensitivity.

**Conclusion:**

This circRNA-Sig model, validated across two large cohorts, offers a novel, clinically actionable tool for stratifying NSCLC patients for atezolizumab therapy, potentially enhancing personalized treatment strategies.

## Introduction

Lung cancer remains the most prevalent malignant tumor and the leading cause of cancer-related mortality worldwide, with non-small-cell lung cancer (NSCLC) accounting for over 85% of cases.^1,2^ Despite advances in clinical management, the 5-year overall survival (OS) rate for NSCLC has only increased from 15% to 25%.^3^ Immune checkpoint inhibitors (ICIs), such as PD-1 and PD-L1 inhibitors, have revolutionized NSCLC treatment by blocking the PD-1/PD-L1 interaction, thereby enhancing T-cell-mediated tumor killing.^4^ Additionally, Chimeric Antigen Receptor T-cell therapy has emerged as a promising immunotherapeutic approach, involving the genetic modification of T cells to express receptors that target specific tumor antigens.^5^ Overall, ICIs are now a standard therapy for advanced NSCLC lacking driver mutations. However, the objective response rate (ORR) to ICI therapy in unselected NSCLC patients ranges from just 10% to 30%.^6,7^ Some patients experience accelerated disease progression or early death after receiving ICI treatment.^8^ In contrast, for patients who have been preliminarily screened, such as those with PD-L1 positive NSCLC, the ORR to ICI therapy reached 30%–45%, whereas it is only 15%–25% in PD-L1 negative patients.^9–11^ Current biomarkers such as PD-L1 expression and tumor mutation burden (TMB) have had a significant impact on clinical decision-making; however, their reliability as markers still has certain limitations. For instance, pre-analytical factors, including sample collection methods, specimen processing, and the types of analyses conducted, can all impact the results.^12^ Moreover, gene mutations have recently been recognized as valuable tools for identifying patients likely to benefit from immunotherapy, complementing the predictive power of PD-L1 and TMB. For example, patients with KRAS mutations experience a significantly improved prognosis following immunotherapy, and among these patients, those with high PD-L1 expression derive even greater benefit. However, in patients with co-mutations in KRAS, KEAP1, and STK11, even those with high PD-L1 expression, the efficacy of immunotherapy remains suboptimal.^13^ Recent studies have shown that these conventional biomarkers can only explain approximately 60% of ICI responses, suggesting that up to 40% of the factors influencing ICI outcomes remain to be discovered.^14^ These results highlighting the urgent need for more reliable biomarkers. Identifying patients likely to benefit from ICI therapy remains a critical challenge in clinical practice, necessitating the discovery of novel, stable predictors to guide personalized treatment strategies.

Circular RNAs (circRNAs) are a class of non-coding RNAs with a closed-loop structure, conferring high stability and resistance to degradation. Though less abundant in the body, they play crucial roles in gene regulation, including transcription, posttranscriptional processing, and translation.^15^ In recent years, with the development of the next-generation sequencing technology, numerous studies have highlighted the significance of circRNAs in NSCLC progression, positioning them as promising candidates for liquid biopsy biomarkers.^16^ Meanwhile, emerging evidence suggests that circRNAs regulate the tumor immune microenvironment across various cancers, thereby affecting the efficacy of immunotherapy.^17^ For example, we identified and validated a panel of five circRNAs in melanoma for the first time. This panel demonstrated strong predictive performance across training, internal validation, and external validation cohorts.^18^ Similarly, Dong et al.^19^ explored the potential of circRNAs as predictive biomarkers for immunotherapy in melanoma patients. Their findings highlight that, compared to currently available biomarkers, the circRNA-based model offers a more effective approach for selecting patients likely to respond to immunotherapy.

Despite these advances, the role of circRNAs as predictive biomarkers for immunotherapy in NSCLC remains largely unexplored. To address this gap, we leveraged The Cancer CircRNA Immunome Atlas (TCCIA), a comprehensive circRNA database for tumor immunotherapy we developed to integrate circRNA profiles with immunotherapy outcomes.^20^ Using RNA sequencing data from 891 advanced NSCLC patients (439 receiving immunotherapy and 452 receiving chemotherapy (CT)) in the OAK (*n* = 699, 344 receiving immunotherapy and 355 receiving chemotherapy) and POPLAR (*n* = 192, 95 receiving immunotherapy and 97 receiving chemotherapy) cohorts accessed via TCCIA, we performed differential expression analysis to identify circRNAs with significant differences in patients treated with ICIs.^21,22^ We then constructed and validated predictive models using machine learning, followed by screened key circRNAs and explored the potential mechanisms affecting NSCLC immunotherapy within the tumor microenvironment context. Our research delivers a robust circRNA based model for predicting the efficacy of immunotherapy in NSCLC, offering a stable and effective biomarker to enhance clinical decision-making.

## Materials and methods

The reporting of this study conforms to the Transparent Reporting of a multivariable prediction model for Individual Prognosis or Diagnosis + artificial intelligence statement and the respective checklist has been provided as a Supplemental File 1.^23^

### Study cohort

The patient clinical characteristics and RNA-seq data for the phase III randomized controlled trial OAK (NCT02008227)^21^ and phase II POPLAR (NCT01903993)^22^ used in this study were sourced from TCCIA database (https://shiny.hiplot.cn/TCCIA/).^20^ In both trails, atezolizumab (anti-PD-L1) was used as the immunotherapy treatment for patients, and the CT control group was treated with docetaxel. This study included all available NSCLC cohorts from the TCCIA database, comprising a total of 891 patients (699 from the OAK cohort and 192 from the POPLAR cohort), including 439 patients receiving immunotherapy (344 from OAK and 95 from POPLAR) and 452 patients receiving CT (355 from OAK and 97 from POPLAR). We selected 439 patients who received ICI for the study. Among them, 344 patients from the OAK cohort were randomly assigned in a 7:3 ratio as the model training set and internal validation set, while 95 patients from the POPLAR cohort were assigned as the external validation set.

### CircRNA identification

We established a novel circRNA identification pipeline based on four independent circRNA detection tools: CIRCexplorer2, CIRIquant, find_circ, and circRNA_finder.^24–27^ With the human reference genome hg38 as reference, circRNAs were identified, parsed, and annotated for each sample. To ensure the biological reliability and robustness of the circRNA data, the following stringent criteria were applied: (1) only circRNAs located on autosomes and sex chromosomes were retained; (2) circRNAs were required to overlap with gene regions defined in the genome annotation file; (3) each circRNA had to be detected by at least two of the four tools, with at least one tool identifying no fewer than two back-splicing junction reads. The quantification results obtained from circRNA identification pipeline are available from the TCCIA database.^20^

### Identification of differentially expressed circRNAs

To identify circRNAs capable of serving as predictive biomarkers for NSCLC immunotherapy, we adapted principles from the concept form BEST (Biomarkers, EndpointS, and other Tools).^28^ Specifically, within the OAK cohort, we performed univariate survival analysis for each circRNA separately in patients receiving ICI and those receiving CT. For each circRNA, patients were stratified into high-expression and low-expression groups using the median expression level as the threshold. We then assessed whether there were significant differences in survival time between these high- and low-expression groups. This process served as an initial screening to identify circRNAs potentially associated with ICI response. The criteria for this initial selection were as follows: (1) A significant association with survival (univariate analysis *p* < 0.05) in the immunotherapy-treated patient group; (2) No significant association with survival (univariate analysis *p* > 0.05) in the CT-treated patient group; (3) The circRNA can be detected (nonzero expression) in more than 5% of the samples. Subsequently, the circRNAs passing this initial screening underwent further selection using LASSO regression analysis to isolate those with the most significant predictive potential.^29^

### Development of predictive models

Using the selected circRNAs, we plan to perform analyses employing several models, including the Cox proportional hazards model, the time-dependent Cox model, Random Forest (RF), support vector machine (SVM), and XGBoost. Given the typically low abundance of circRNAs in patient samples and the inherent variability in detection across specimens, clinical interpretation often prioritizes the mere detectability of a circRNA over its specific expression level. Consequently, patients in whom a circRNA is detectable are generally considered distinct from those in whom it is undetectable, regardless of the quantified expression level. Therefore, in addition to developing models based on the continuous expression values of circRNAs, we will also explore an alternative approach involving the binarization of circRNA expression data. Specifically, patients with detectable circRNA expression will be assigned a value of 1, while those with undetectable expression will be assigned a value of 0.

For the training set, models will be developed using both the continuous and binarized expression data across all five modeling techniques. Subsequently, the median output score from each model will serve as a threshold to stratify patients into high-risk and low-risk groups. Kaplan–Meier analysis will then be performed to assess whether there are significant differences in prognosis between these stratified groups. Concurrently, the predictive performance of the models over time will be evaluated using time-dependent area under the curve (AUC) values. The predictive efficacy of these models will be further validated using both internal and external validation sets. Ultimately, the model demonstrating the optimal combination of stability and accuracy across these validation steps will be established as the final ICI prediction model.

To evaluate the predictive performance of our model, we gathered several existing scoring models that utilize transcriptome-based features to predict ICI efficacy. We then calculated and compared the 6-, 12-, and 18-month AUC values for these established models and our proposed model within the training, internal validation, and external validation set.

### Gene set enrichment analysis

We obtained the 50 hallmark gene sets collection from The Molecular Signatures Database (MSigDB, http://software.broadinstitute.org/gsea/msigdb/).^30^ This collection comprises gene sets representing well-established biological pathways. We then performed Gene Set Enrichment Analysis (GSEA) using this collection as a reference to compare patients stratified by high versus low model scores.^31^ The objective was to determine the activation or suppression patterns of these canonical pathways between the two groups. Furthermore, to investigate differences in immune cell infiltration between the high- and low-score groups, we extracted characteristic gene sets for 22 tumor-infiltrating immune cell types (LM22) from CIBERSORT.^32^ We conducted GSEA using these LM22 gene sets as the reference. Concurrently, we employed the Gene Set Variation Analysis (GSVA) algorithm to calculate enrichment scores for each of the cell types.^33^ By integrating the findings from both GSEA and GSVA, we aimed to identify specific immune cell populations associated with the model score. To further explore more detailed subtypes of immune cells, GSEA and GSVA analyses were conducted based on the B-cell subtype gene set proposed by Ma et al.^34^ and the T-cell subtype gene set proposed by Chu et al.^35^

### Statistical analysis

The Cox proportional hazards model was applied to estimate hazard ratios (HRs) and 95% confidence intervals (CIs) between each group, and the two-sided log-rank test was used for survival analysis among each groups, with the significance-level alpha = 0.05. Analysis was conducted in R version 4.4.3. The package we utilize as follows: the “VennDiagram” package (version 1.7.3), the “ggplot2” package (version 3.5.2), the “dplyr” package (version 1.1.4), the “survival” package (version 3.8.3), the “survminer” package (version 0.5.0), the “timeROC” package (version 0.4), the “randomForestSRC” package (version 3.3.3), the “xgboost” package (version 1.7.10.1), the “survivalsvm” package (version 0.0.6), the “clusterProfiler” package (version 3.16.1), the “org.Hs.eg.db” package (version 2.4.6).

## Results

### CircRNA detection and distribution in NSCLC

**Figure 1.**
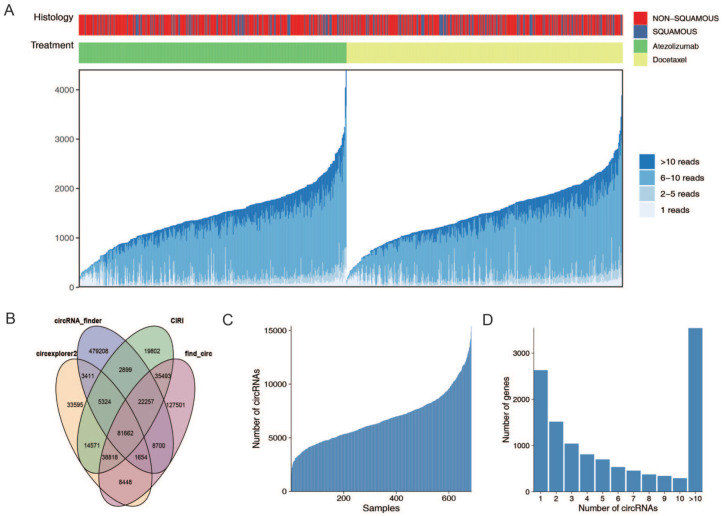
Characterization of circRNAs in OAK cohort. CircRNAs identified from 699 NSCLC patients in the OAK cohort through the circRNA identification pipeline (a). The *x*-axis indicates patients and the y-axis indicates the number of circRNAs identified. The number of circRNAs recognized by each circRNA recognition tool in the OAK cohort, a total of 81,662 circRNAs in the 699 patients of the OAK cohort was identified (b). The number of circRNAs identified for each sample (c) and circRNAs corresponding to host genes (d). Most host genes were associated with more than 10 distinct circRNAs. circRNA, circular RNAs; NSCLC, non-small cell lung cancer.

This study included 699 patients from the OAK cohort (344 received ICIs) and 192 patients from the POPLAR cohort (95 received ICIs), and collected patient characteristics are shown in Table 1. To enhance the reliability of circRNA identification, we utilized our previously established circRNA identification pipeline, which jointed results from four circRNA detection tools including circexplorer2, circRNA_finder, CIRI, and find_circ.^24–27^ In result, we identified a total of 81,662 distinct circRNAs in the 699 patients of the OAK cohort (Figure 1(a) and (b); see “Materials and methods” section for details). We next examined the number of circRNAs detected per patient. To minimize potential bias, circRNAs detected in fewer than 20% of patients were excluded. Following this filtering step, the number of detected circRNAs per patient primarily ranged from 1536 to 15,408 (Figure 1(c)). Further analysis of the corresponding host genes revealed that a total of 12,232 genes are involved and most host genes were associated with more than 10 distinct circRNAs (Figure 1(d)). Similarly, 66,400 circRNAs were identified in the 192 patients of the POPLAR cohort and the number of circRNAs that can be detected in each patient ranges from 1359 to 24,860. There are a total of 11,224 genes involved, and same as OAK cohort, most host genes were associated with more than 10 distinct circRNAs (Supplemental Figure 1).

**Table 1.** Patient characteristics.

Factors	OAK (*n* = 699)		POPLAR (*n* = 192)	
	Atezolizumab (*n* = 344)	Docetaxel (*n* = 355)	Atezolizumab (*n* = 95)	Docetaxel (*n* = 97)
Sex				
Male	219(63.66%)	222(62.54%)	68(71.58%)	53(54.64%)
Female	125(36.34%)	133(37.46%)	27(28.42%)	44(45.36%)
Histology				
Squamous	87(25.29%)	103(29.01%)	36(37.89%)	36(37.11%)
Non-squamous	257(74.71%)	252(70.99%)	59(62.11%)	61(62.89%)
Response				
Complete Response (CR)	4(1.16%)	0(0.00%)	1(1.05%)	0(0.00%)
Partial Response (PR)	44(12.79%)	42(11.83%)	12(12.63%)	16(16.49%)
Stable Disease (SD)	111(32.27%)	158(44.51%)	40(42.11%)	35(36.08%)
Progressive Disease (PD)	159(46.22%)	115(32.39%)	34(35.79%)	35(36.08%)

### Identification of circRNAs associated with immune responses

**Figure 2.**
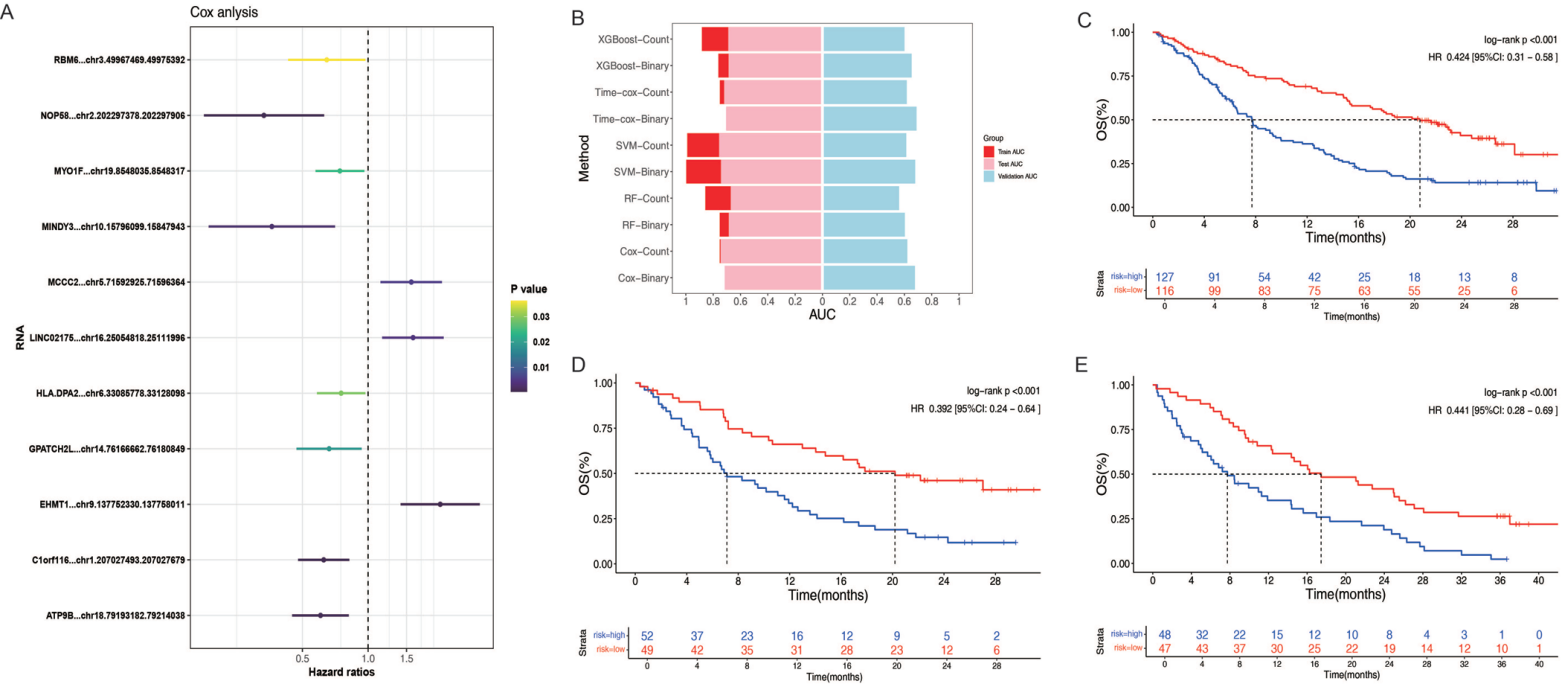
Construction and validation of circRNA-Sig based model. Forest plot of HR for OS of selected 11 circRNAs related to immunotherapy for NSCLC, including 3 risk factors and 8 protective factors (a). Establish predictive models for circRNA-Sig using multiple methods and validate their efficacy (b), Binary-Cox model was ultimately chosen as the prediction model. Patients were divided into high-risk and low-risk groups based on the median predicted risk score of model. Survival analysis was conducted on the two groups in the training set (c), internal validation set (d), and external validation set (e), respectively. The results showed that patients in the low-risk group had significantly longer survival times compared to those in the high-risk group. circRNA, circular RNA; HR, hazard ratio; NSCLC, non-small cell lung cancer; OS, overall survival.

Served the median expression level of each circRNA as the threshold, patients were stratified into high- and low-expression groups. Univariate analysis was subsequently employed to assess significant differences between these groups (see “Materials and methods” for details). This initial screening yielded 294 circRNAs from a pool of 81,662 candidates from training cohort. To further refine this selection and identify the most significant circRNAs, LASSO regression analysis was conducted, ultimately pinpointing 11 circRNAs, named circRNA-Sig (Figure 2(a)). These included three circRNAs identified as risk factors (HR > 1) and eight identified as protective factors (HR < 1). Most of them have not been reported in previous studies, but most of the host genes have been shown to have biological functions in various tumors (Supplemental Table 1). For instance, *MCCC2*, the host gene of *circMCCC2*, has been shown to be overexpressed in various cancer types, where it acts as an oncogene. In colorectal cancer, *MCCC2* expression is significantly elevated in tumor tissues compared to adjacent normal tissues, and cellular experiments have confirmed that *MCCC2* overexpression promotes tumor cell proliferation, invasion, and migration.^36^ Similarly, *MCCC2* is upregulated in hepatocellular carcinoma, correlating with poor prognosis; this effect may be mediated through the upregulation of leucine metabolism pathways crucial for tumor survival.^37^ Furthermore, a correlation analysis of the expression levels of these 11 circRNAs revealed no significant intercorrelations, supporting their use as independent variables in subsequent modeling (Supplemental Figure 2).

Based on circRNA-Sig, we developed prediction models using five distinct approaches, including Cox model, time-dependent Cox, RF, SVM, and XGBoost. The predictive performance of each model was subsequently evaluated in both internal and external validation cohorts, and corresponding survival curves were generated. The results revealed that, irrespective of the model employed, patients stratified into high-risk and low-risk groups based on the median risk score exhibited significantly different prognoses. After comprehensively comparing the AUC values across the different datasets, we selected the Binary-Cox model as the final predictive model due to its robust and effective predictive performance (AUC_train = 0.71, AUC_internal = 0.72, AUC_external = 0.68; Figure 2(b)). Survival analysis indicated that patients with high-risk scores had significantly shorter survival times compared to those with low-risk scores in the training cohort (HR = 0.424, 95% CI: 0.31–0.58, *p* < 0.001, Figure 2(c)). This finding was consistently observed in both the internal validation cohort (HR = 0.392, 95% CI: 0.24–0.64, *p* < 0.001, Figure 2(d)) and the external validation cohort (HR = 0.441, 95% CI: 0.28–0.69, *p* < 0.001, Figure 2(e)).

**Figure 3.**
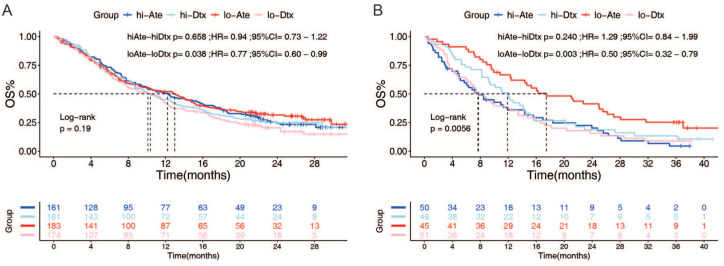
CircRNA-Sig based model play roles as predictive biomarker. Patients in the OAK (a) and POPLAR (b) cohorts were grouped by treatment and risk score. The results showed that patients in the low-risk group who received immunotherapy had significantly longer survival time than those who received chemotherapy, while there was no significant difference in the high-risk group. circRNA, circular RNA.

To further investigate the association between the risk score and ICI response, we analyzed all patients within the OAK cohort. Patients were stratified into four groups based on their risk score (low vs high) and treatment modality (CT vs ICI): low-risk/CT, low-risk/ICI, high-risk/CT, and high-risk/ICI. Survival analysis revealed no significant difference in survival outcomes between patients receiving ICI and those receiving CT within the high-risk group (HR = 0.94, 95% CI: 0.73–1.22, *p* = 0.658, Figure 3(a)). However, among patients in the low-risk group, those treated with ICI demonstrated significantly better prognoses compared to those treated with CT (HR = 0.77, 95% CI: 0.60–0.99, *p* = 0.038, Figure 3(a)). These findings suggest that the risk score may function as a predictive biomarker for ICI benefit. Specifically, patients with low-risk scores appear more likely to derive greater benefit from ICI compared to CT. This conclusion was further validated in the POPLAR cohort, where consistent results indicated that patients with low-risk scores were more likely to benefit from ICI (HR = 0.50, 95% CI: 0.32–0.79, *p* = 0.003, Figure 3(b)).

### CircRNA-Sig presents a superior transcriptomic-based model compared to previous approaches

**Figure 4.**
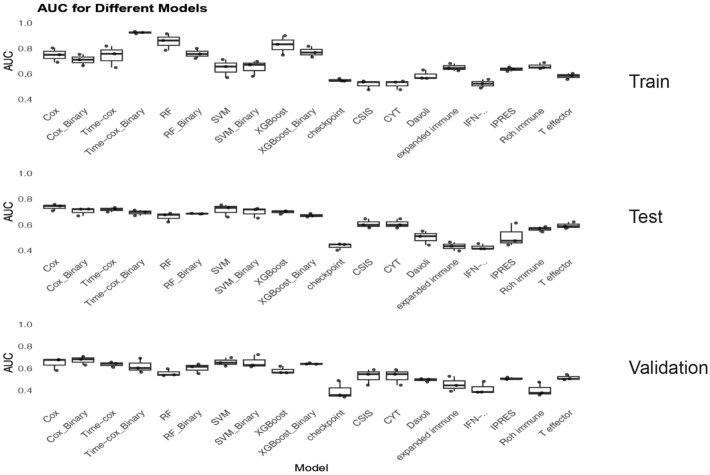
Comparisons of predictive models and other transcriptome-based signatures. For predictive models based on circRNA-Sig and other transcriptome-based signatures, we calculate the AUC values at 6, 12, and 18 months on the training set, internal validation set, and external validation set, respectively. Regardless of which model is based on circRNA-Sig, the predictive performance is superior to other signatures. AUC, area under the curve; circRNA, circular RNA.

Prior research has investigated the prediction of patient response to ICI using transcriptomic features, leading to the development of several predictive models. To validate the predictive performance of the features identified in our study, we gathered nine previously published predictive models developed using distinct transcriptomic features, most of which were obtained by calculating the average value of gene expression log10 conversion (Supplemental Table 2).^31,38–44^ We then calculated the respective AUC values for each model at 6, 12, and 18 months across the training, internal validation, and external validation sets (Figure 4). The results showed that, with the exception of our final Binary–Cox model, the other predictive models developed using the circRNAs we identified achieved AUC values ranging from 0.67 to 0.92 across the training, internal validation, and external validation sets. In contrast, the nine previously published models demonstrated comparatively poorer performance across these three datasets, yielding AUC values ranging from 0.52 to 0.68. Notably, there is several overlap in these transcriptomic-based models, such as the *GZMB* present in five models, but the transcriptome features of all models do not overlap with the host genes of circRNA-Sig. These findings suggest that, specifically within the context of NSCLC patients, the circRNAs identified in our study may offer consistent superior predictive capability for patient response to ICI compared to existing transcriptomic features known for strong predictive performance in other cancer types or pan-cancer cohorts.

### Active immune microenvironment enriched in low circRNA-sig scored patient group

**Figure 5.**
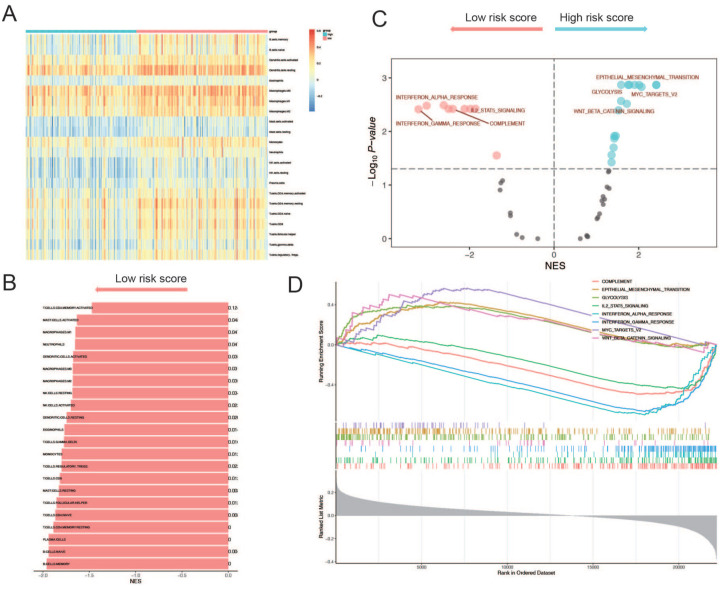
Function exploration of circRNA-Sig. Based on the LM22 cell gene set provided by CIBERSORT, GSEA (a) and GSVA (b) were performed on high-risk and low-risk patients, respectively. The immune microenvironment of low-risk patients is generally more active than that of high-risk patients. Perform GSEA analysis on 50 hallmark gene sets (c, d), in low-risk patients, pathways that activate the immune environment are significantly enriched, while in high-risk patients, pathways that promote tumor progression are significantly enriched. circRNA, circular RNA; GSEA, Gene Set Enrichment Analysis; GSVA, Gene Set Variation Analysis.

To provide a more comprehensive and reliable research model for predicting the potential mechanisms of ICI efficacy from the perspectives of intergroup enrichment analysis and single sample gene set activity evaluation, we performed GSEA and GSVA using the CIBERSORT LM22 cell gene set. The results indicated that patients with low-risk scores exhibited a more active immune microenvironment compared to those with high-risk scores (Figure 5(a) and (b)). Further analysis of immune cell subtypes suggested that both T cell and B cell lineages were generally more active in patients with low-risk scores, particularly the T cell lineage (Supplemental Figure 3). GSEA of common canonical pathways revealed significant enrichment in pathways associated with enhanced immune function in patients with low risk scores. Notably, the interferon (IFN)-α and IFN-γ pathways were enriched. Previous studies have shown that the interferon family plays a critical role in driving PD-L1 expression in both cancer and host cells, with IFN-γ being a key cytokine produced by activated T cells and natural killer (NK) cells within the tumor microenvironment.^38,45^ The enrichment of these two pathways suggests that patients with low-risk scores have a more active immune microenvironment, which may lead to a better response to immunotherapy. The IL2/STAT5 pathway has a dual effect in immunotherapy, on the one hand, activation of the IL-2/STAT5 pathway can activate immune cells such as CD8+T cells and NK cells, on the other hand, IL2/STAT5 can cause CD8+ T-cell exhaustion^46,47^ (Figure 5(c) and (d)).

Conversely, pathways promoting oncogenic processes, such as the Wnt/β-Catenin pathway and the epithelial-mesenchymal transition (EMT) pathway, were significantly enriched in patients with high-risk scores (Figure 5(c) and (d)). The latest research shows that abnormal activation of Wnt/β-catenin signaling pathway promotes cancer cell escape from immune surveillance and inhibits T cell infiltration, leads to poor prognosis of immunotherapy in patients.^48^ The EMT pathway is a key regulatory factor for tumor cell invasion and metastasis, significantly contributing to the fatal progression of the disease.^49^ This suggests that patients with low-risk scores tend to have more active immune systems, whereas those with high-risk scores are more prone to tumor progression. These findings align with our previous analyses and potentially represent the underlying mechanism enabling our model to predict ICI response, but actual role of these pathways in NSCLC patients still needs further research.

## Discussion

The role of circRNAs in cancer is garnering increasing attention. However, research investigating the link between circRNAs and cancer immunotherapy remains in the exploratory phase. In this study, we systematically characterized the differential expression profiles of circRNAs in NSCLC patients treated with ICIs, utilizing data from two independent cohorts. Concurrently, we identified a panel of 11 circRNAs associated with immunotherapy efficacy, named circRNA-Sig. Subsequently, we employed machine-learning methods to construct a circRNA-based predictive model for ICI response in these patients. This study presents the first circRNA signature prediction model and systematic analysis for immune therapy in NSCLC. Importantly, it employed two independent large clinical cohorts for analysis and validation, ensuring the robustness and stability of the results. CircRNA-Sig model offers a novel approach for prospectively identifying patients likely to benefit from immunotherapy in clinical practice.

Numerous previous studies have highlighted the critical roles of circRNAs in the pathogenesis and progression of NSCLC. However, the precise relationship and underlying mechanisms between circRNAs and tumor immunotherapy still require further investigation. While most of the circRNAs in the circRNA-Sig have not been extensively studied in the context of NSCLC, some circRNAs that have been explored in other diseases still provide valuable insights for our study.

For instance, circGPATCH2L, which we hypothesize plays a protective role in NSCLC, has been reported in a study by Chen et al.^50^ to promote cell apoptosis by binding to TRIM28 as a protein decoy in the nucleus pulposus. This mechanism, observed in intervertebral disc cells, suggest that circGPATCH2L protective role in NSCLC tumor tissue, acting as a tumor-suppressive factor by modulating apoptotic pathways.

Moreover, circATP9B, which is also considered a protective factor in NSCLC, has been shown to promote cell apoptosis by upregulating PTEN expression in patients with ulcerative colitis.^51^ This finding suggests that circATP9B play a similar role in NSCLC by regulating apoptotic mechanisms, although its exact function in the context of NSCLC remains unclear.

However, some previously reported biological functions of circRNAs differ from our findings. For example, circEHMT1, which we identified as a risk factor in NSCLC, has been shown in breast cancer studies to inhibit cancer cell metastasis by reducing MMP2 levels through the circEHMT1/miR-1233-3p/KLF4 axis.^52^ In breast cancer, this mechanism is thought to contribute to a more favorable prognosis by controlling metastatic spread. The difference in circEHMT1’s role across different cancer types highlights the complexity of circRNA function and suggests that its behavior be context-dependent.

Additionally, many different circRNAs share the same host gene, making it challenging to interpret their distinct roles. To address this, we conducted further data collection and analysis on the reported circEHMT1. Although the article did not directly provide the sequence or chromosomal localization of circEHMT1, we compared the forward and reverse primers used in the study with the circEHMT1 sequence in circRNA-Sig and found that the two circRNAs refer to different molecules. This emphasizes the need for further research to clarify the true mechanisms of circRNAs in circRNA-Sig, as different circRNAs from the same host gene have distinct biological effects in different cancer contexts.

Due to the high stability of circRNA, it can be detected not only in tumor tissues but also in peripheral blood. Recent studies have identified three serum exosomal circRNAs for early diagnosis of NSCLC by sequencing blood samples from patients, and a diagnostic kit based on these circRNAs has been developed.^53^ However, the main challenges in using blood to detect circRNA are its low expression levels in blood and interference from circRNAs originating from normal tissues. Currently, the main detection methods for circRNA can be divided into de novo identification and targeted identification. The most commonly used targeted identification method is qRT-PCR, but it faces issues such as high costs and the lack of standardized internal reference genes, which hinder its clinical application.^17^ Additionally, the NanoString nCounter technology, which counts individual circRNA transcripts using fluorescent probes, has been tested in some cancers and shows promise for further clinical application.^54^ Although circRNA shows good potential as a biomarker in many studies, its clinical application remains limited due to technical challenges in detection. Similarly, cfRNA liquid biopsy has also faced issues of low expression levels and low sensitivity in blood. Recently, a new method for cfRNA liquid biopsy, RARE-seq, has been proposed. By utilizing a dual design of Random Priming and Affinity Capture, the sensitivity of detecting tumor-derived cfRNA was increased by approximately 50 times, with a detection limit of 0.05%.^55^ Therefore, the future development of circRNA can follow a similar path to cfRNA, focusing on the identification of tumor-specific circRNAs while advancing high-sensitivity detection technologies to promote its clinical application.

While circRNAs have emerged as prognostic biomarkers in cancer patients, their potential as predictive biomarkers for immunotherapy efficacy remains to be explored. Compared with other transcriptome-based signatures, our predictive model demonstrated strong predictive performance across two distinct NSCLC immunotherapy cohorts. However, these findings stem entirely from retrospective analyses, and further prospective studies are warranted to validate our results. Currently, immunotherapy is often used in combination with other treatments in clinical practice, such as immunotherapy combined with chemotherapy, neoadjuvant immunotherapy prior to surgery, and immunotherapy combined with radiotherapy. However, the two cohorts included in this study are limited to the use of monotherapy with immunotherapy. Future studies will incorporate more diverse cohorts to validate the predictive efficacy of this model and further explore the functional role of circRNAs. In addition, patients with advanced NSCLC are often accompanied by multiple concomitant diseases. Due to limitations in the data sources, this study was unable to collect information regarding concomitant diseases. However, it is undeniable that concomitant diseases are an important factor influencing treatment strategies in cancer. The relationship between circRNAs, cancer treatment, and concomitant diseases remains to be further explored.

## Conclusion

In conclusion, this study systematically analyzed circRNA expression profiles across two NSCLC clinical studies, identified differentially expressed circRNAs, and subsequently developed a predictive model for immunotherapy response in NSCLC patient, which holds promise for guiding clinical treatment.

## Supplemental Material

sj-docx-6-tam-10.1177_17588359251395920 – Supplemental material for CircRNA signature predicts immunotherapy response in advanced non-small cell lung cancer Supplemental material, sj-docx-6-tam-10.1177_17588359251395920 for CircRNA signature predicts immunotherapy response in advanced non-small cell lung cancer by Xin Li, Shixiang Wang, Yanru Cui, Su-Han Jin, Junzhu Xu, Chi Zhang, Juanyan Shen, Hu Ma and Jian-Guo Zhou in Therapeutic Advances in Medical Oncology

sj-jpg-1-tam-10.1177_17588359251395920 – Supplemental material for CircRNA signature predicts immunotherapy response in advanced non-small cell lung cancer Supplemental material, sj-jpg-1-tam-10.1177_17588359251395920 for CircRNA signature predicts immunotherapy response in advanced non-small cell lung cancer by Xin Li, Shixiang Wang, Yanru Cui, Su-Han Jin, Junzhu Xu, Chi Zhang, Juanyan Shen, Hu Ma and Jian-Guo Zhou in Therapeutic Advances in Medical Oncology

sj-jpg-2-tam-10.1177_17588359251395920 – Supplemental material for CircRNA signature predicts immunotherapy response in advanced non-small cell lung cancer Supplemental material, sj-jpg-2-tam-10.1177_17588359251395920 for CircRNA signature predicts immunotherapy response in advanced non-small cell lung cancer by Xin Li, Shixiang Wang, Yanru Cui, Su-Han Jin, Junzhu Xu, Chi Zhang, Juanyan Shen, Hu Ma and Jian-Guo Zhou in Therapeutic Advances in Medical Oncology

sj-jpg-3-tam-10.1177_17588359251395920 – Supplemental material for CircRNA signature predicts immunotherapy response in advanced non-small cell lung cancer Supplemental material, sj-jpg-3-tam-10.1177_17588359251395920 for CircRNA signature predicts immunotherapy response in advanced non-small cell lung cancer by Xin Li, Shixiang Wang, Yanru Cui, Su-Han Jin, Junzhu Xu, Chi Zhang, Juanyan Shen, Hu Ma and Jian-Guo Zhou in Therapeutic Advances in Medical Oncology

sj-xlsx-4-tam-10.1177_17588359251395920 – Supplemental material for CircRNA signature predicts immunotherapy response in advanced non-small cell lung cancer Supplemental material, sj-xlsx-4-tam-10.1177_17588359251395920 for CircRNA signature predicts immunotherapy response in advanced non-small cell lung cancer by Xin Li, Shixiang Wang, Yanru Cui, Su-Han Jin, Junzhu Xu, Chi Zhang, Juanyan Shen, Hu Ma and Jian-Guo Zhou in Therapeutic Advances in Medical Oncology

sj-xlsx-5-tam-10.1177_17588359251395920 – Supplemental material for CircRNA signature predicts immunotherapy response in advanced non-small cell lung cancer Supplemental material, sj-xlsx-5-tam-10.1177_17588359251395920 for CircRNA signature predicts immunotherapy response in advanced non-small cell lung cancer by Xin Li, Shixiang Wang, Yanru Cui, Su-Han Jin, Junzhu Xu, Chi Zhang, Juanyan Shen, Hu Ma and Jian-Guo Zhou in Therapeutic Advances in Medical Oncology

## Declarations

**Ethics approval and consent to participate:** This study has been approved by the Medical Ethics Review Committee of the Second Affiliated Hospital of Zunyi Medical University (KYLL-2022-004). This study was conducted in accordance with the ethical principles of the Helsinki Declaration. This study does not apply the ‘Consent to Participate’ statement.

**Consent for publication:** Not applicable.

**Availability of data and materials:** Researchers can use the manuscript-related data on the Cancer Circular RNA Immunology Database (TCCIA, https://shiny.hiplot.cn/TCCIA/) for analysis. Raw manuscript-related data are available upon reasonable request. Requests can be made to jianguo.zhou@zmu.edu.cn. Codes were implemented in R 4.4.1 and are deposited in https://github.com/OncoHarmony-Network/CircRNA-analysis.

**ORCID iDs:** Xin Li  https://orcid.org/0009-0004-0253-4097 Jian-Guo Zhou  https://orcid.org/0000-0002-5021-3739
