# Supplementary figures and images for: CircRNA signature predicts immunotherapy response in advanced non-small cell lung cancer
Source: Ther Adv Med Oncol. 2025 Nov 25;17:17588359251395920. doi: 10.1177/17588359251395920 (PMC12647551; doi:10.1177/17588359251395920)

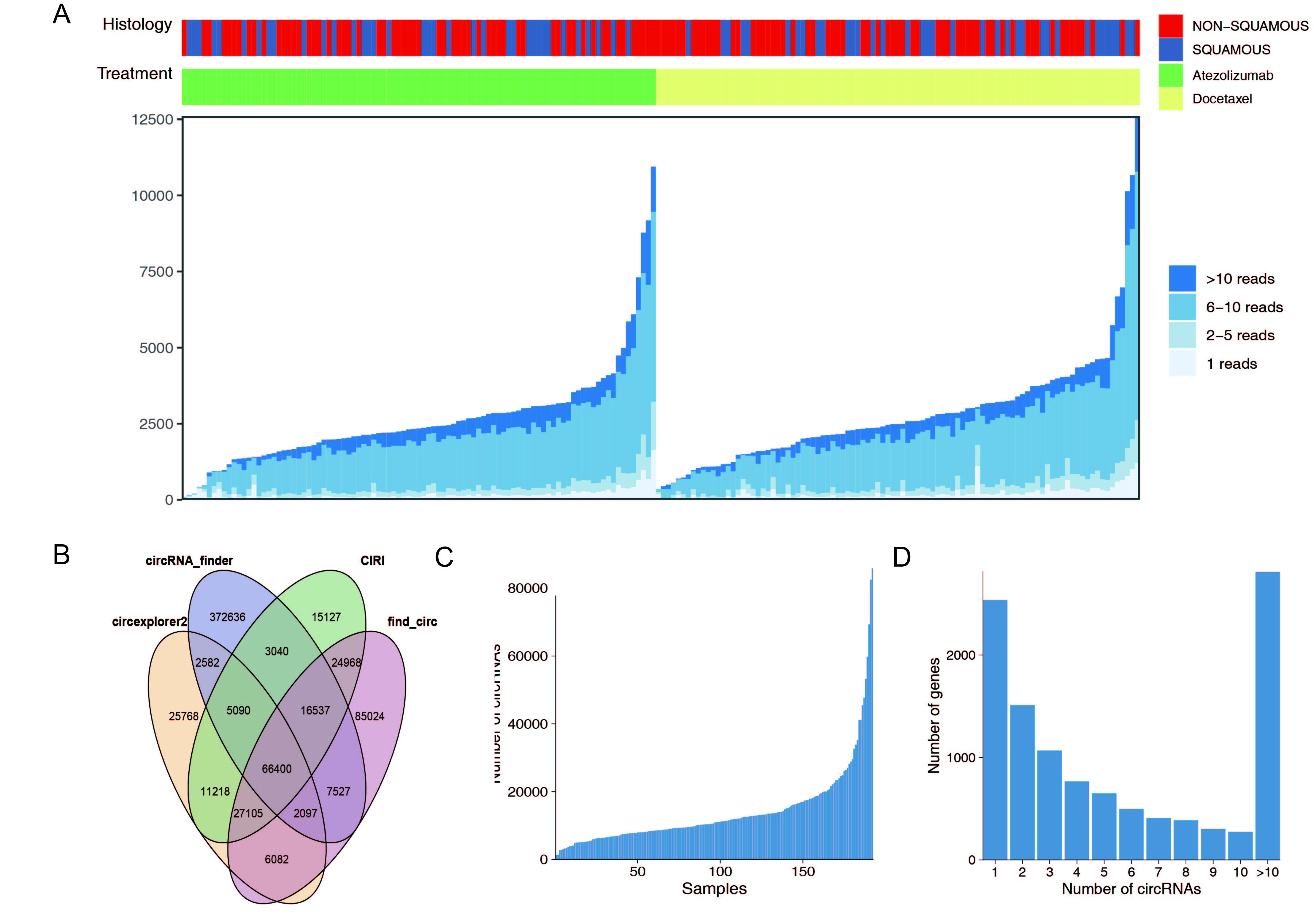

Supplement: sj-jpg-1-tam-10.1177_17588359251395920 – Supplemental material for CircRNA signature predicts immunotherapy response in advanced non-small cell lung cancer [file sj-jpg-1-tam-10.1177_17588359251395920.jpg]

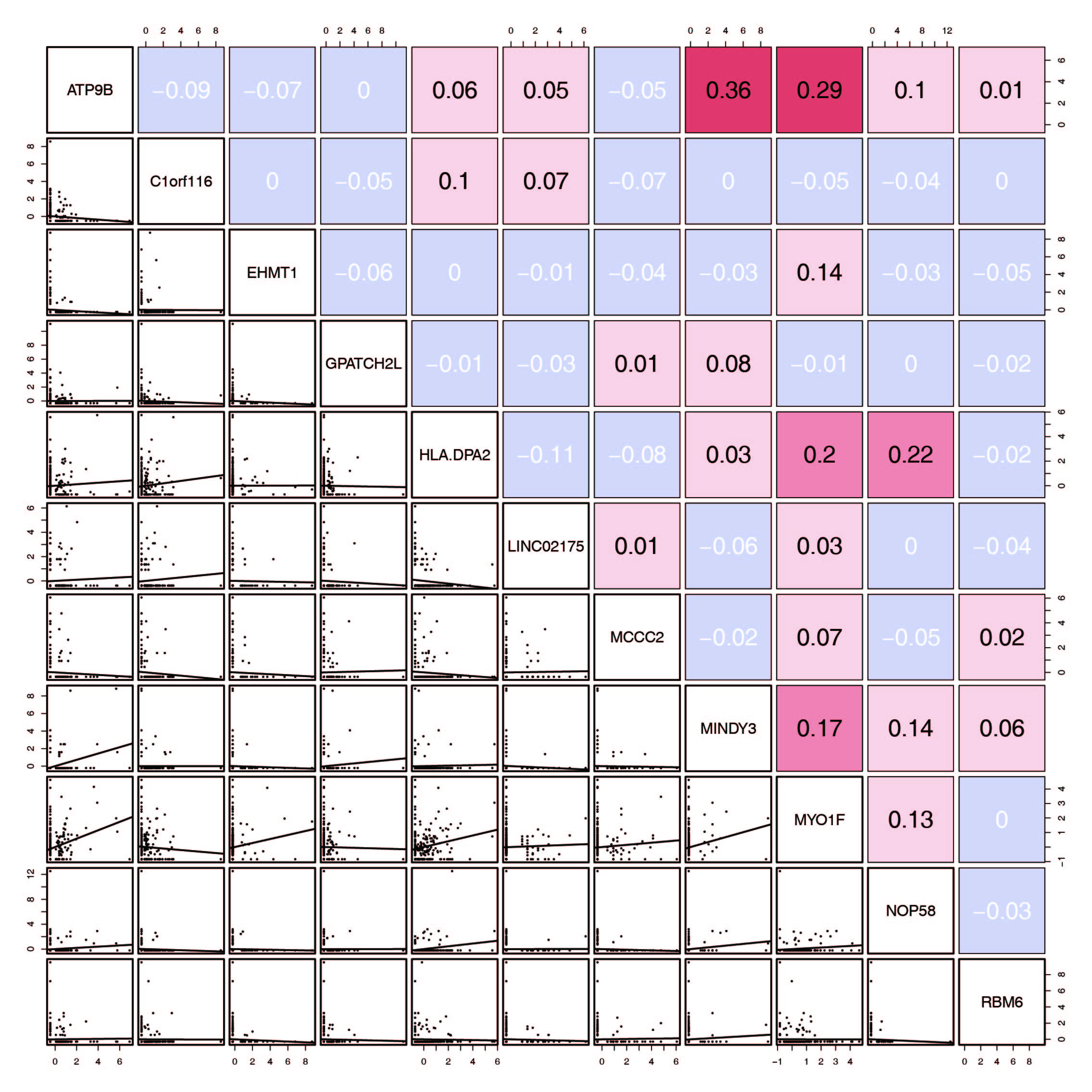

Supplement: sj-jpg-2-tam-10.1177_17588359251395920 – Supplemental material for CircRNA signature predicts immunotherapy response in advanced non-small cell lung cancer [file sj-jpg-2-tam-10.1177_17588359251395920.jpg]

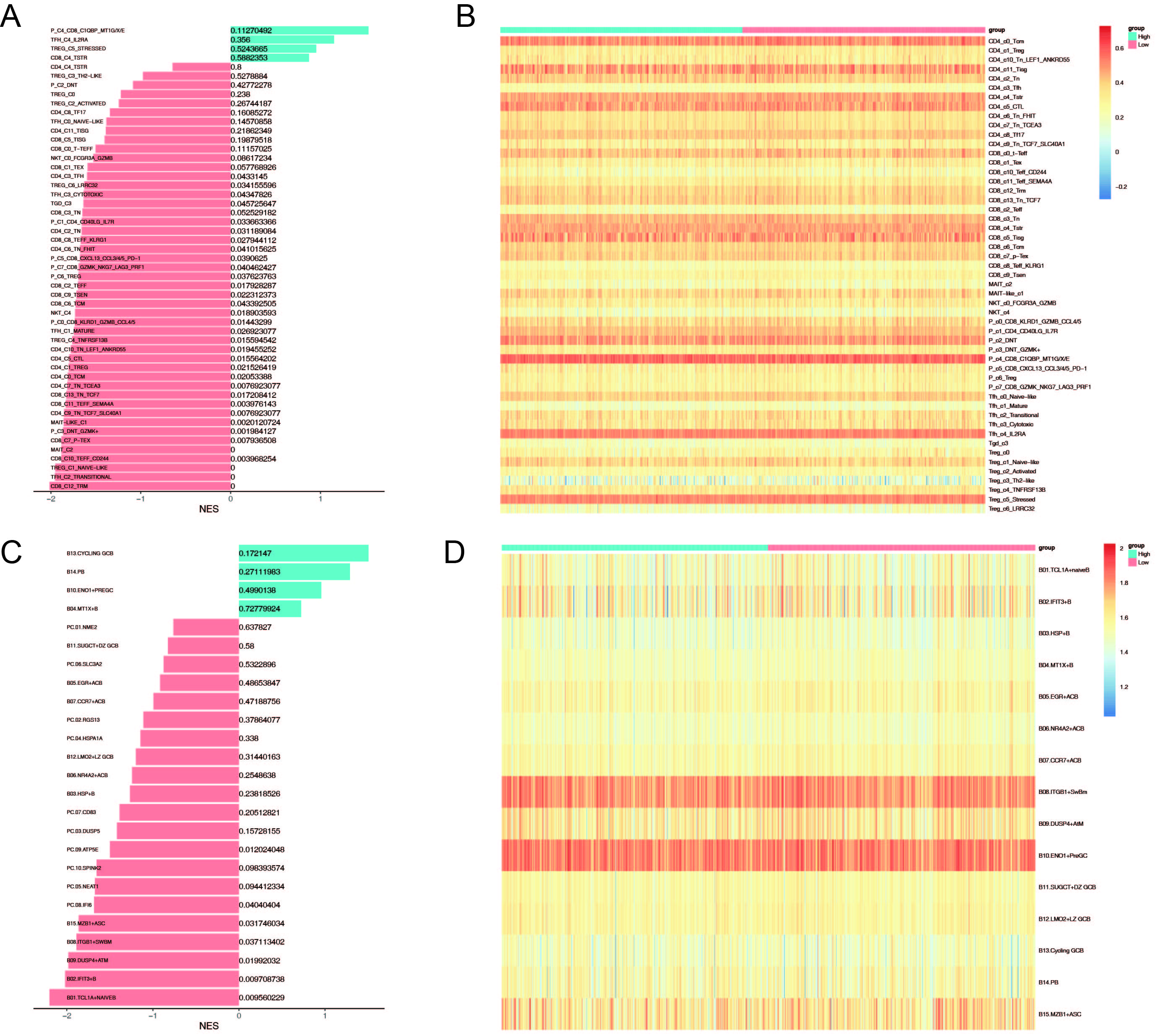

Supplement: sj-jpg-3-tam-10.1177_17588359251395920 – Supplemental material for CircRNA signature predicts immunotherapy response in advanced non-small cell lung cancer [file sj-jpg-3-tam-10.1177_17588359251395920.jpg]
